# Thrombin Aptamer-Modified Metal–Organic Framework Nanoparticles: Functional Nanostructures for Sensing Thrombin and the Triggered Controlled Release of Anti-Blood Clotting Drugs

**DOI:** 10.3390/s19235260

**Published:** 2019-11-29

**Authors:** Wei-Hai Chen, Ola Karmi, Bilha Willner, Rachel Nechushtai, Itamar Willner

**Affiliations:** 1 Institute of Chemistry, Center for Nanoscience and Nanotechnology, The Hebrew University of Jerusalem, Jerusalem 91904, Israel; chenwhwhu@gmail.com (W.-H.C.); bilhawillner@gmail.com (B.W.); 2 Institute of Life Science, The Hebrew University of Jerusalem, Jerusalem 91904, Israel; olakarmi@yahoo.com (O.K.); rachel@vms.huji.ac.il (R.N.)

**Keywords:** Factor Xa, switch, nanomedicine, DNA-nanotechnology, sensor

## Abstract

This paper features the synthesis of thrombin-responsive, nucleic acid-gated, UiO-68 metal–organic framework nanoparticles (NMOFs) loaded with the drug Apixaban or rhodamine 6G as a drug model. Apixaban acts as an inhibitor of blood clots formation. The loads in the NMOFs are locked by duplex nucleic acids that are composed of anchor nucleic acids linked to the NMOFs that are hybridized with the anti-thrombin aptamer. In the presence of thrombin, the duplex gating units are separated through the formation of thrombin–aptamer complexes. The unlocking of the NMOFs releases the drug (or the drug model). The release of the drug is controlled by the concentration of thrombin. The Apixaban-loaded NMOFs revealed improved inhibition, as compared to free Apixaban, toward blood clot formation. This is reflected by their longer time intervals for inducing clot formation and the decreased doses of the drug required to affect clots formation. The beneficial effects of the Apixaban-loaded NMOFs are attributed to the slow-release mechanism induced by the NMOFs carriers, where the inhibition of factor Xa in the blood clotting cycle retards the formation of thrombin, which slows down the release of the drug.

## 1. Introduction

Metal–organic framework nanoparticles (NMOFs) represent a broad class of porous nanostructures [1,2,3,4]. The NMOFs are formed by the crystallization of metal ions in the presence of appropriate stoichiometry of organic ligands. Different applications of the porous NMOFs were suggested, including their use for gas separation [5] and purification [6], frameworks for catalytic transformations [7,8,9,10], sensors [11,12], drug carriers [13,14,15,16], and functional materials for fuel cells [17,18].

Recently, drug-loaded nucleic acid-gated nano/microcarriers have attracted substantial interest as stimuli-responsive drug carriers [19,20,21,22,23]. Different drug-loaded stimuli-responsive nucleic acid-functionalized carriers such as SiO_2_ nanoparticles [19], liposomes [24], microcapsules [25], and hydrogels [26] were prepared, and the triggered release of the loaded drugs from the carriers were demonstrated. Different triggers, such as pH [26], light [27], heat [28], catalytic nucleic acids [29], chemical reagents [30], and aptamer–ligand complexes [31], were used to unlock the carriers and release the loads. Within these broad efforts, the use of nucleic acid-gated, drug-loaded, metal–organic framework nanoparticles (NMOFs) acting as stimuli-responsive carriers [32,33] was recently demonstrated by our laboratory. We have shown that the drug-loaded NMOFs can be capped by stimuli-responsive nucleic acid gates. The nucleic acid gating units were unlocked in the presence of pH [30], enzymes [29], DNAzymes [30], miRNA [32], and the formation of aptamer–ligand complexes [33], leading to the release of the drug. In addition, the effective permeation of the drug (doxorubicin)-loaded NMOFs into cells was achieved, and selective cytotoxicity of the drug-loaded NMOFs toward cancer cells, in the presence of cancer cells biomarkers (e.g., miRNAs) or environmental cancer cell conditions, e.g., acidic pH, over-expressed ATP, or vascular endothelial growth factor (VEGF), were demonstrated. The promising therapeutic applications of drug-loaded NMOFs calls for the search of other applications of such stimuli-responsive carriers in nanomedicine. Aptamers are sequence-specific nucleic acids that specifically bind low-molecular-weight substrates or macromolecules, e.g., proteins. The specific binding properties of aptamers were extensively used to develop optical [34], electrochemical [35], photoelectrochemical [36], and piezoelectric sensors [37]. Particularly, the anti-thrombin aptamer was applied as a recognition matrix for developing different thrombin-sensing configurations [38,39]. This basic background was used to develop the thrombin-responsive Apixaban-loaded NMOFs for inhibiting blood clotting. The treatment of venous thromboembolism is challenging among patients subjected to a high-risk incomplete protection of thrombotic activity [40]. Apixaban is an oral inhibitor of factor Xa (FXa) that plays a central role in the blood-clotting cascade [41]. Apixaban is a common drug for treating thromboembolic disorders, such as minimizing the risk of strokes. 

Here, we report on the preparation of thrombin-responsive drug-loaded NMOFs for the inhibition of clot formation. Thrombin, being a central component in the blood clotting machinery, triggers the unlocking of the gated NMOFs through the formation of the thrombin–aptamer complexes and stimulates the release of the anti-clotting drug, Apixaban. The released drug Apixaban cooperatively inhibits the blood-clotting cascade (Apixaban inhibits factor Xa (FXa) in the clot cascade) [42]. That is, the stimuli-responsive NMOFs acts as functional thrombin sensors that lead to the autonomous unlocking of the gated NMOFs, which is a process that activates the release of the anti-clotting drug. Thus, the study utilizes the porous structures of NMOFs for the effective loading of the anti-blood clotting Apixaban drug. In addition, we combine the thrombin aptamer units in the NMOFs as functional stimuli-responsive gates that allow the thrombin-triggered release of the anti-blood clotting drug from the NMOFs carriers. As a result, we present a means to control the anti-blood clotting process by the natural blood clotting cycle.

## 2. Experimental

Materials: Thrombin, vascular endothelial growth factor 165 human (VEGF), hemoglobin, and bovine serum albumin (BSA) were purchased from Sigma-Aldrich. Ultrapure water was obtained by a NANOpure Diamond instrument (Barnstead International, Dubuque, IA, USA). All nucleic acid strands were provided by Integrated DNA Technologies Inc. (Coralville, IA, USA). The detailed DNA sequences used in the present study are:

(**1**) 5′-NH_2_-(CH_2_)_6_-AAAACCAACCA-3′;
(**2**) 5′-GGTTGGTGTGGTTGGTTTT-3′.

The synthesis of azide-functionalized NMOFs (NMOF-N_3_): Firstly, the organic ligand, amino-triphenyldicarboxylic acid, was prepared according to a reported method [43]. Afterwards, NMOFs were synthesized by reacting 42 mg of ZrCl_4_ with 60 mg of amino-triphenyldicarboxylic acid in 100 mL at 80 °C for 5 days. The resulting NMOFs were collected by centrifugation and washed with *N*,*N*-dimethylformamide (DMF), triethylamine/ethanol (1:20, V/V), and ethanol, respectively. For the preparation of azide-modified NMOFs (NMOF-N_3_), 8 mg of the obtained NMOFs were dispersed in 4 mL of tetrahydrofuran (THF), followed by adding 1.5 mL of the tert-butyl nitrite (tBuONO) and 1.2 mL of the azidotrimethylsilane (TMSN_3_). Then, the reaction mixture was stirred at room temperature overnight to obtain NMOF-N_3_ nanoparticles.

The preparation of dibenzocyclooctyne (DBCO)-functionalized nucleic acid (**1**) (DBCO-DNA): To modify nucleic acid (**1**) with the DBCO functional group, 100 μL of nucleic acid (**1**) (1 mM) was reacted with 150 μL of dibenzocyclooctyne-sulfo-*N*-hydroxysuccinimidyl ester (DBCO-sulfo-NHS) (6 mM) in 4-(2-hydroxyethyl)piperazine-1-ethanesulfonic acid (HEPES) buffer (10 mM, pH = 7.4), and the reaction mixture was vigorously shaken overnight. Thereafter, the solution was filtered with MicroSpin G-25 columns (GE-Healthcare) to separate any unreacted DBCO-sulfo-NHS and to obtain the pure DBCO-DNA.

Preparation of the nucleic acid (**1**)-functionalized NMOFs: To synthesize nucleic acid (**1**)-functionalized NMOFs, NMOF-N_3_ nanoparticles (5 mg, 2.5 mL) were reacted with DBCO-modified nucleic acid (**1**) (100 nmol, 1 mL). The mixture was incubated at 40 °C for 72 h, and then three portions of NaCl were added to the reaction mixture every two hours within the first 6 h to reach a final concentration of 0.5 M. Thereafter, the obtained nucleic acid (**1**)-functionalized NMOFs were washed three times with HEPES buffer (10 mM, pH = 7.4) to remove unbound DNA. The UV absorbance of the wash was measured at 260 nm to evaluate the amount of DNA loading on the NMOFs.

Loading of nucleic acid (**1**)-functionalized NMOFs: The nucleic acid (**1**)-functionalized NMOFs, 2 mg, were incubated with rhodamine 6G (0.5 mg/mL) or with the anticoagulant drug, Apixaban (0.5 mg/mL) overnight in 1.5 mL of HEPES buffer solution (10 mM, pH = 7.4). Subsequently, the NMOFs were separated and transferred to a HEPES buffer solution (10 mM, pH = 7.4) that contained NaCl, 20 mM. Then, the NMOFs were hybridized with the nucleic acid (**2**), leading to the locked state of the duplex DNA-functionalized NMOFs loaded with Apixaban or rhodamine 6G. After 12 h, the resultant NMOFs were washed several times to remove the excess and nonspecifically bound rhodamine 6G or Apixaban.

Thrombin-induced unlocking of the NMOFs and the release of the encapsulated loads: Experiments were performed using solutions of the respective rhodamine 6G or Apixaban-loaded (**1**)/(**2**)-locked NMOFs at a concentration corresponding to 1 mg/mL. Then, the NMOFs solutions, 30 μL, were treated with 10 μL of variable concentrations of thrombin for a fixed time-interval of 30 minutes. Other proteins, e.g. VEGF, hemoglobin, and BSA, were used as controls to demonstrate the selective unlocking of the NMOFs by thrombin. After incubation, the respective samples were centrifuged at 10,000 rpm for 10 min to precipitate the NMOFs, and the fluorescence of the released loads in the supernatant solution was measured using a Cary Eclipse Fluorescence Spectrophotometer (Varian Inc., Palo Alto, CA, USA).

Apixaban activity detection on normal platelet-poor plasma (PPP): Pooled normal platelet-poor plasma (PPP) has been prepared as described in a previous study [44]. PPP have been incubated either with empty NMOFs, or with free Apixaban (1.08, 3.2, and 5.4 nM), respectively, or with empty NMOFs along with free Apixaban of the previous determined concentrations, or NMOFs loaded with the Apixaban of the previous same concentrations. Then, clot formation assay have been initiated by using CaCl_2_ (15 µM) at room temperature with continuous shaking. The onset of clot formation was the time determined as the inflection point before turbidity increases, as described in a previous study [45].

## 3. Results and Discussion

**Figure 1:**
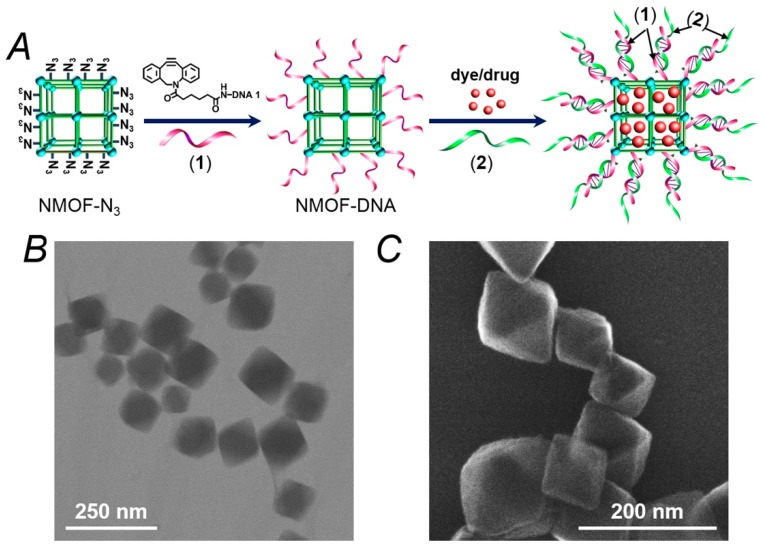
(**A**) Schematic synthesis of the nucleic acid (**1**)-functionalized UiO-68 metal–organic framework nanoparticles (NMOFs) loaded with a dye (rhodamine 6G) or a drug (Apixaban) and gated with the thrombin-responsive duplexes (**1**)/(**2**). (**B**) TEM image of the (**1**)-modified NMOFs. (**C**) SEM image of the (**1**)-functionalized NMOFs.

Figure 1A depicts the synthesis of the thrombin-responsive UiO-68 nucleic acid-gated NMOFs loaded with the Apixaban drug or rhodamine 6G as drug model. The UiO-68 NMOFs were prepared by mixing Zr^4+^ ions with the amino-triphenyldicarboxylic acid ligand. The amine functionalities were transformed into azide groups, and the dibenzocyclooctyne (DBCO)-modified with the nucleic acid (**1**) was coupled to the azide groups, using click chemistry principles to yield the (**1**)-modified NMOFs. The loading of the nucleic acid (**1**) was evaluated spectroscopically, and it corresponded to 9.4 nmol/mg of NMOFs. Brunauer–Emmett–Teller (BET) measurements revealed that a surface area of the NMOFs corresponded to ca. 1220 m^2^/g and a pore size of 1.58 nm. The nucleic acid (**1**) complements partially the base sequence of the thrombin aptamer [46,47]. Figure 1B,C show the TEM and SEM images of the nucleic acid (**1**)-functionalized NMOFs. Bipyramidal nanoparticles in the size range of 100–150 nm are observed. The (**1**)-modified NMOFs interacted with a solution of Apixaban or rhodamine 6G to allow the adsorption of the drug or drug model on the porous NMOFs. The drug (or drug model)-loaded (**1**)-modified NMOFs were reacted with the thrombin aptamer (**2**), and the resulting duplexes (**1**)/(**2**) provided the locks for the entrapment of the loads in the NMOFs. It should be noted that the modification of the NMOFs with a foreign nucleic acid that does not hybridize with the aptamer-containing sequence, (**2**), does not allow the formation of dye/drug-loaded NMOFs that include protecting gating units. (The loads are immediately washed off from the NMOFs). In addition, the hybridization of a non-aptamer-containing sequence with the foreign nucleic acids yields dye/drug-loaded NMOFs that do not respond to thrombin. These results indicate the selectivity of the (**1**)/(**2**)-gated NMOFs for the specific thrombin-guided release of the loads.

**Figure 2:**
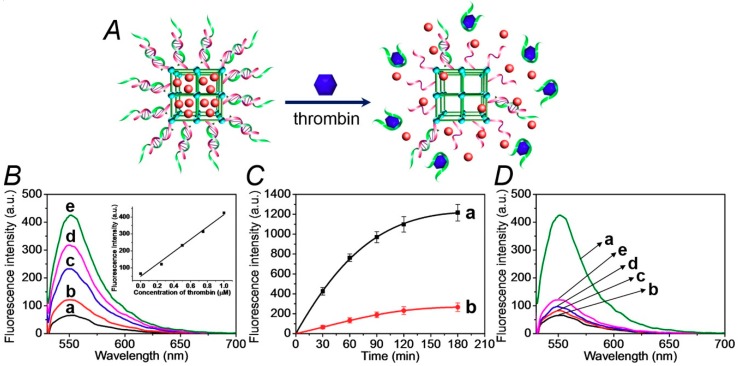
(**A**) Schematic unlocking of the (**1**)/(**2**)-gated NMOFs and the release of rhodamine 6G, in the presence of thrombin, via the separation of the duplex gates by the formation of aptamer/thrombin complexes. (**B**) Fluorescence spectra of the released rhodamine 6G from the dye-loaded NMOFs upon treatment with variable concentrations of thrombin for a fixed time-interval of 30 minutes: (a) 0 μM, (b) 0.25 μM, (c) 0.5 μM, (d) 0.75 μM, and (e) 1.0 μM. Inset: the calibration curve of the released rhodamine 6G upon treatment with different concentrations of thrombin. (**C**) Time-dependent fluorescence changes upon the release of rhodamine 6G from the (**1**)/(**2**)-gated dye-loaded NMOFs in the presence of: (a) thrombin, 1 μM, (b) No thrombin added. (**D**) Fluorescence spectra of rhodamine 6G released from the (**1**)/(**2**)-gated, dye-loaded, NMOFs upon treatment for a fixed time interval corresponding to 30 minutes with: (a) thrombin, 1 μM, (b) no thrombin added, (c) bovine serum albumin (BSA), 1 μM, (d) hemoglobin, 1 μM, (e) vascular endothelial growth factor (VEGF), 1 μM. The binding of the thrombin to the aptamer units and all the release experiments were performed in the HEPES buffer solution, 10 mM, pH = 7.4, containing 20 mM of NaCl, 10 mM of KCl, and 10 mM of MgCl_2_.

The thrombin-stimulated release of the drug-model rhodamine 6G is schematically presented in Figure 2A. The association of thrombin to the aptamer strand (**2**) releases the aptamer–thrombin complex from the NMOFs. The separation of the duplex units (**1**)/(**2**) unlocks the NMOFs, resulting in the release of the loads. Figure 2B shows the fluorescent spectra of the released rhodamine 6G after a fixed time interval corresponding to 30 minutes, upon subjecting the NMOFs to variable concentrations of thrombin. As the concentration of thrombin increases, the release of rhodamine 6G is enhanced, consistent with the higher degree of thrombin-induced unlocking of the NMOFs. Figure 2B, inset, shows the resulting calibration curve that corresponds to the release of rhodamine 6G in the presence of variable concentrations of thrombin, indicating that the release of the fluorophore from the NMOFs provides an effective optical means to sense thrombin. Figure 2C, curve (a), shows the time-dependent fluorescence changes corresponding to the released rhodamine 6G from the NMOFs subjected to thrombin, 1 µM. After a time-interval of ca. 180 minutes, the release process levels off to a saturation level, implying that the release of the fluorescent dye was completed. Using an appropriate calibration curve, we estimate that ca. 57.3 nmol of rhodamine 6G were released from one milligram of the NMOFs. Figure 2B, curve (b), shows the fluorescence changes of the released rhodamine 6G from the loaded NMOFs in the absence of added thrombin. A very inefficient release of rhodamine 6G is observed and this levels off after ca. 180 min. This inefficient release of the fluorophore is attributed to the release of rhodamine 6G bound to incompletely locked or defective sites of the NMOFs. Figure 2C shows the selective release of the load driven by the formation of the thrombin–aptamer complex. In these experiments, we challenged the (**1**)/(**2**)-gated, rhodamine 6G-loaded NMOFs with foreign proteins, e.g., BSA, hemoglobin, VEGF. We demonstrate that only thrombin unlocks the (**1**)/(**2**)-gated NMOFs and releases the fluorescent load. With all foreign proteins or in the absence of thrombin, only the background inefficient release of the load is observed.

**Figure 3:**
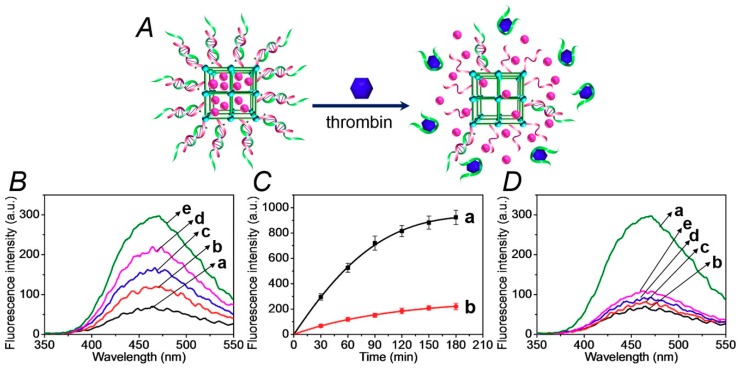
(**A**) Schematic unlocking of the (**1**)/(**2**)-gated NMOFs and the release of Apixaban, in the presence of thrombin, via the separation of the duplex gates by the formation of aptamer-thrombin complexes. (**B**) Fluorescence spectra of the released Apixaban from the drug-loaded NMOFs upon treatment with variable concentrations of thrombin for a fixed time interval of 30 minutes: (a) 0 μM, (b) 0.25 μM, (c) 0.5 μM, (d) 0.75 μM, and (e) 1.0 μM. (**C**) Time-dependent fluorescence changes upon the release of Apixaban from the (**1**)/(**2**)-gated drug-loaded NMOFs in the presence of (a) thrombin, 1 μM, or (b) no thrombin added. (**D**) Fluorescence spectra of Apixaban released from the (**1**)/(**2**)-gated, drug-loaded, NMOF upon treatment for a fixed time interval corresponding to 30 minutes with (a) thrombin, 1 μM; (b) no thrombin added; (c) BSA, 1 μM; (d) hemoglobin, 1 μM; and (e) vascular endothelial growth factor (VEGF), 1 μM.

In the next step, the thrombin-stimulated release of the Apixaban anti-blood clotting drug is evaluated, as shown in Figure 3. In this system, the Apixaban-loaded (**1**)/(**2**)-locked NMOFs were subjected to thrombin. The unlocking of the gating units by the dissociation of the locks, via the formation of the thrombin/aptamer (**2**) complexes, is shown in Figure 3A, resulted in the release of the drug. Figure 3B shows the fluorescence spectra of the released Apixaban upon reacting the drug-loaded NMOFs with variable concentrations of thrombin for a fixed time interval corresponding to 30 minutes. As the concentration of thrombin increases, the release of the drug is enhanced, which is consistent with the increased unlocking of the (**1**)/(**2**) gating units. Figure S1 shows the concentrations of Apixaban released from the NMOFs after a fixed time interval of 30 minutes, upon subjecting the NMOFs to variable concentrations of thrombin. The release rate reaches a saturation value at a thrombin concentration that corresponds to 4 µM, implying that at this concentration of thrombin, all of the aptamer gating units are unlocked. Figure 3C, curve (a), depicts the time-dependent fluorescence changes of the released Apixaban upon treatment of the (**1**)/(**2**)-locked NMOFs with thrombin, 1 µM. The release rate of the drug reaches a saturation value after ca. 180 minutes, which is the time interval required for the complete release of the loaded drug. From the saturated fluorescence value of the released drug, and using an appropriate calibration curve, we estimated the loading of the NMOFs with the drug to be 32.5 nmol/mg of NMOFs. For comparison, Figure 3C, curve (b), depicts the release profile of the Apixaban from the loaded NMOFs in the absence of added thrombin. An inefficient release of the drug proceeds that levels off after ca. 150 min, which is presumably from NMOFs sites that include imperfect locking units. Figure 3D shows the fluorescence spectra of the released Apixaban, after a fixed time interval of the (**1**)/(**2**)-locked NMOFs interacting with thrombin, 1 µM, or a series of foreign proteins. The release of the drug in the presence of the foreign proteins shows only the background release of the drug, which is similar to the release profile of the drug in the absence of thrombin. The results demonstrate that (i) the thrombin selectively unlocks the Apixaban-loaded NMOFs, and (ii) the drug-release process correlates with the concentration of thrombin; as the concentration of thrombin is higher, the release of the drug is enhanced. These results suggest that there is an intimate control over the blood-clotting cascade, and particularly control over the function of thrombin and factor Xa in the blood-clotting cascade. Namely, the increased concentration of thrombin activates the release of the Apixaban, which inhibits factor Xa. Then, the inhibition of FXa leads to the decreased formation of thrombin and to the inhibition of the blood-clotting cascade. It should be noted that after the release of the model dye (rhodamine 6G) or of the thrombin-induced release of Apixaban, the TEM/SEM images did not show any structural morphology changes, and the particles could be reloaded with the respective dye/drug components. Furthermore, the release patterns of the re-loaded nanoparticles were very similar to the release curves observed in the first cycles, implying that the NMOFs retained their structural feature. These experiments indicate that the NMOFs can be recycled as functional carriers.

**Figure 4:**
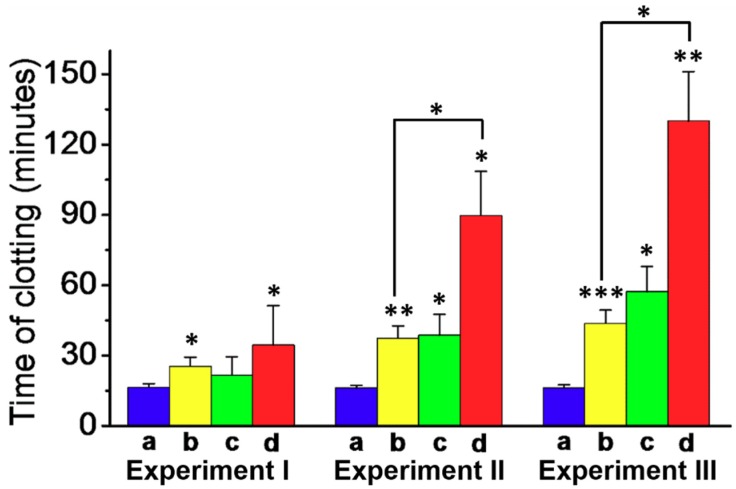
Apixaban-induced inhibition of clot formation on normal platelet-poor plasma using the Apixaban-loaded (**1**)/(**2**)-gated NMOFs loaded with different Apixaban concentrations and control systems: (a) Blue, the unloaded (**1**)/(**2**)-gated NMOFs. (b) Yellow, the pure Apixaban drug. (c) Green, the unloaded NMOFs and free Apixaban. (d) Red, the Apixaban-loaded (**1**)/(**2**)-gated NMOFs. Experiment I, Concentration of loaded/free Apixaban, 1.08 nM. Experiment II, Concentration of loaded/free Apixaban, 3.2 nM. Experiment III, Concentration of Apixaban, 5.4 nM. Clot formation was stimulated using 15 μM of CaCl_2_, incubated at room temperature under continuous shaking. Results derived from three different experiments. * *p* < 0.05, ** *p* < 0.001, and *** *p* < 0.001.

In the next step, the effect of the Apixaban-loaded (**1**)/(**2**)-locked NMOFs on the clot formation in normal platelet-poor plasma (PPP) was examined, as shown in Figure 4. The assay for the preparation of the PPP samples and the method to evaluate the time intervals for clot formation are detailed in the experimental section. In these experiments, we examined the effects of three concentrations of Apixaban in the absence of NMOFs and the presence of the drug-loaded NMOFs on clot formation, while introducing appropriate control systems, as shown in panels I, II, III. Analysis of these results reveals that the drug-loaded (**1**)/(**2**)-gated NMOFs have an obvious efficacy effect on the clot formation time intervals. The results allow deriving the following conclusions. (i) The concentration of Apixaban affects the time interval of the formation of the clots. For example, the samples treated with pure Apixaban 1.08 nM and 5.4 nM yielded clot formation after ca. 25 min and 45 min, respectively. (ii) The unloaded NMOFs have no effect on clot formation. (iii) The unloaded NMOFs and free Apixaban reveal similar clot formation activities to those of pure Apixaban. (iv) The drug-loaded NMOFs reveal superior anti-clot formation activities as compared to free Apixaban. At a drug concentration corresponding to 3.2 nM, the clot formation by free Apixaban is about 40 min, while the drug-loaded NMOFs, at the same drug concentration, lead to a substantially longer clot-formation time interval of ca. 90 min. Similarly, at a drug concentration corresponding to 5.4 nM, the pure Apixaban induces clot formation within 45 min, whereas the drug-loaded NMOFs lead to clot formation after a time interval of ca. 120 min. (v) It is interesting to note that the Apixaban-loaded NMOFs at a concentration of 3.2 nM reveal a substantial prolonged clot formation time interval (90 min), as compared to the time interval of clot formation in the presence of the free drug at a high concentration of 5.4 nM (45 min). Thus, the application of Apixaban-loaded NMOFs could significantly reduce the doses of the drug to reach beneficial therapeutic functions. The superior functions of the Apixaban-loaded NMOFs can be attributed to the slow-release feature of the drug from the NMOFs (Cf. Figure 4, Green Columns). It should be noted that the thrombin-stimulated release of a foreign drug, e.g. camptothecin, did not induce any inhibition in clot formation, suggesting that the clot formation is selective for Apixaban or other blood clotting drugs.

## 4. Conclusions

In conclusion, the present article described the assembly of NMOFs loaded with the Apixaban, a drug, inhibiting blood clot formation, which are gated by the anti-thrombin aptamer. The drug-loaded NMOFs were unlocked by thrombin, and this resulted in the release of Apixaban. The thrombin-induced controlled release of the drug from the NMOFs revealed superior functions, as compared to free Apixaban, at similar concentrations toward clot formation. The thrombin–aptamer-gated NMOFs loaded with the factor Xa-inhibiting drug demonstrated unique autonomous functions for inhibiting clot formation by the blood-clotting cascade. The NMOFs act as a thrombin-sensing unit and as the concentration of thrombin increased, the release of Apixaban was enhanced. Nonetheless, the release of the drug inhibits the formation of thrombin via the blood-clotting cascade, leading to the slowdown of the release of the inhibitor. That is, the functionalized NMOFs demonstrate an autonomous dose-controlled release of the drug. The similar concept may be applied to design thrombin aptamer-gated NMOFs for other anti-blood clotting drugs. It would be interesting to compare an Apixaban-induced inhibition of clot formation in the absence and presence of the NMOFs to other assays probing the Apixaban-stimulated inhibition of clot formation. Table S1, supporting information, summarizes the evaluation of the Apixaban-induced inhibition of clot formation by different assays and using different doses of the drug. Particularly interesting is the comparison of our system to the assay probing clot formation by light scattering (follows the fibrinolysis by factor Xa) [48], entry 2 in Table S1. Our results clearly indicate that Apixaban-loaded NMOFs reveal a significantly prolonged (ca. three-fold) time interval to induce clotting, as compared to free Apixaban. In addition, the dose of Apixaban-induced inhibition of clot formation is ca. 80-fold lower than the value determined by the light-scattering assay.

## Supplementary Materials

The following are available online at https://www.mdpi.com/1424-8220/19/23/5260/s1, Figure S1: Fluorescence spectra of the released Apixaban from the drug-loaded NMOFs upon treatment with variable concentrations of thrombin for a fixed time-interval of 30 min. The release rate reaches a saturation value at a thrombin concentration that corresponds to 4 µM, implying that at this concentration of thrombin all aptamer gating units are unlocked, Table S1: Comparison of Apixaban-induced inhibition of clot formation.
