# Supplementary material for: Thrombin Aptamer-Modified Metal–Organic Framework Nanoparticles: Functional Nanostructures for Sensing Thrombin and the Triggered Controlled Release of Anti-Blood Clotting Drugs
Source: Sensors (Basel). 2019 Nov 29;19(23):5260. doi: 10.3390/s19235260 (PMC6929137; doi:10.3390/s19235260)
Supplement: Supplementary file 1 [file sensors-19-05260-s001.pdf]

Thrombin Aptamer-Modified Metal-Organic Framework Nanoparticles: Functional Nanostructures for Sensing Thrombin and the Triggered Controlled Release of Anti-Blood Clotting Drugs

Wei-Hai Chen,[1] Ola Karmi,[2] Bilha Willner,[1] Rachel Nechushtai,[2] and Itamar Willner[1,*]

[1]Institute of Chemistry, Center for Nanoscience and Nanotechnology, The Hebrew University of Jerusalem, Jerusalem 91904, Israel

[2]Institute of Life Science, The Hebrew University of Jerusalem, Jerusalem 91904, Israel

* To whom correspondence should be addressed. Tel: 972-2-6585272. Fax: 972-2-6527715.

E-mail: willnea@vms.huji.ac.il

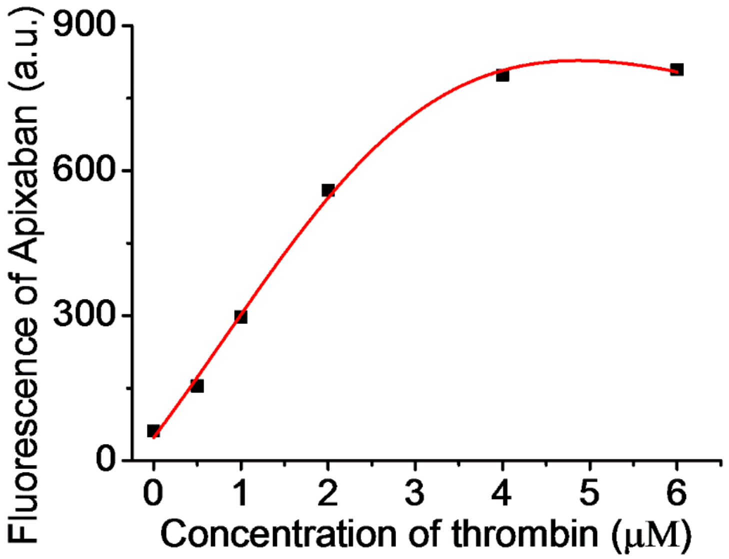

Figure S1. Fluorescence spectra of the released Apixaban from the drug-loaded NMOFs upon treatment with variable concentrations of thrombin for a fixed time-interval of 30 min. The release rate reaches a saturation value at a thrombin concentration that corresponds to 4 μM, implying that at this concentration of thrombin all aptamer gating units are unlocked.

Table S1. Comparison of Apixaban-induced inhibition of clot formation.

Method of detection	Concentration of drug used to induce clot formation	Reference
Monitoring of prothrombinase using fluorophore-labeled thrombin	200 nM[a]	*TH Open* **2018**, *2*, e190-e201.
Probing time-dependent clot formation by light scattering, λ = 405 nm[b]	440 nM<br>ca. 35 minutes to induce clot formation	*J. Thromb. Haemost.* **2018**, *16*, 2276-2288.
Clot waveform analysis used to activate partial thromboplastin	800 nM[c]	*J. Clin. Pathol.* **2019**, *72*, 244-250.
Present study[d]	5.4 nM	

a. No systematic report on the concentrations of Apixaban-induced clot formation is provided.
b. Method follows the fibrinolysis by factor Xa.
c. Time of Apixaban-induced clot formation not stated.
d. Method described in the experimental section.
